# Vitamin D supplementation protects against reductions in plasma 25‐hydroxyvitamin D induced by open‐heart surgery: Assess‐d trial

**DOI:** 10.14814/phy2.14747

**Published:** 2021-02-13

**Authors:** Tyler Barker, Heidi T. May, John R. Doty, Donald L. Lappe, Kirk U. Knowlton, John Carlquist, Kristin Konery, Shannon Inglet, Ben Chisum, Oxana Galenko, Jeffrey L. Anderson, Joseph B. Muhlestein

**Affiliations:** ^1^ Precision Genomics Intermountain Healthcare St. George Utah USA; ^2^ Nutrition and Integrative Physiology University of Utah Salt Lake City Utah USA; ^3^ Heart Institute Intermountain Healthcare Salt Lake City Utah USA; ^4^ School of Medicine University of Utah Salt Lake City Utah USA

**Keywords:** acute stress, cardiovascular, open‐heart surgery patients, Vitamin D

## Abstract

Low vitamin D (serum or plasma 25‐hydroxyvitamin D (25(OH)D)) is a global pandemic and associates with a greater prevalence in all‐cause and cardiovascular mortality and morbidity. Open‐heart surgery is a form of acute stress that decreases circulating 25(OH)D concentrations and exacerbates the preponderance of low vitamin D in a patient population already characterized by low levels. Although supplemental vitamin D increases 25(OH)D, it is unknown if supplemental vitamin D can overcome the decreases in circulating 25(OH)D induced by open‐heart surgery. We sought to identify if supplemental vitamin D protects against the acute decrease in plasma 25(OH)D propagated by open‐heart surgery during perioperative care. Participants undergoing open‐heart surgery were randomly assigned (double‐blind) to one of two groups: (a) vitamin D (*n* = 75; cholecalciferol, 50,000 IU/dose) or (b) placebo (*n* = 75). Participants received supplements on three separate occasions: orally the evening before surgery and either orally or per nasogastric tube on postoperative days 1 and 2. Plasma 25(OH)D concentrations were measured at baseline (the day before surgery and before the first supplement bolus), after surgery on postoperative days 1, 2, 3, and 4, at hospital discharge (5–8 days after surgery), and at an elective outpatient follow‐up visit at 6 months. Supplemental vitamin D abolished the acute decrease in 25(OH)D induced by open‐heart surgery during postoperative care. Moreover, plasma 25(OH)D gradually increased from baseline to day 3 and remained significantly increased thereafter but plateaued to discharge with supplemental vitamin D. We conclude that perioperative vitamin D supplementation protects against the immediate decrease in plasma 25(OH)D induced by open‐heart surgery.

**ClinicalTrials.gov Identifier:** NCT02460211.

## Graphical abstract

Open‐heart surgery induces acute stress and acute stress is detrimental to circulating 25‐hydroxyvitamin D (25(OH)D) concentration. We found that perioperative vitamin D supplementation abolished the decrease in circulating 25(OH)D following open‐heart surgery. Thus, supplemental vitamin D protects against the deleterious impact of acute stress on circulating 25(OH)D concentrations.

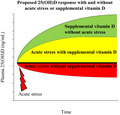

## 1 INTRODUCTION

Low vitamin D (i.e., serum or plasma 25‐hydroxyvitamin D (25(OH)D) concentration) is a global pandemic and associates with a higher prevalence in all‐cause and cardiovascular mortality (Amrein et al., 2014; Dobnig et al., 2008; Sempos et al., 2013). Cardiovascular risk factors (i.e., diabetes, hypertension, hyperlipidemia, and peripheral vascular disease) and complications (i.e., coronary artery disease, myocardial infarction, heart failure, and renal failure) also associate with low or deficient vitamin D levels (Anderson et al., 2010; Zittermann et al., 2013). Low vitamin D levels are prevalent before cardiac surgery (Braun et al., 2014) and associate with major adverse cardiac and cerebrovascular events during inpatient care (Zittermann et al., 2015) and mortality months (Zittermann et al., 2013) after cardiac surgery.

Decreased sun exposure, increased sunscreen use, increasing obesity, and a low amount of vitamin D obtained from dietary sources are widely appreciated as significant contributors to low circulating 25(OH)D concentrations in many regions of the world (Holick, 2007; Norman et al., 2007). Although conflicting evidence exists following myocardial infarction, cardiac surgery, and acute infection (Barth et al., 2012; Newens et al., 2006; Ney et al., 2019), there is general agreement that severe stress lowers circulating 25(OH)D concentrations (Binkley et al., 2017; Duncan et al., 2012; Ghashut et al., 2014). Specifically, circulating 25(OH)D concentrations decrease immediately after orthopedic surgery (Barker et al., 2012; Binkley et al., 2017; Louw et al., 1992; Reid et al., 2011; Waldron et al., 2013), acute pancreatitis (Bang et al., 2011), and cardiac surgery (i.e., coronary artery bypass, cardiac valve replacement, or isolated valve replacement) (Alemzadeh et al., 2012; Borgermann et al., 2012; Canbaz et al., 2008; Krishnan et al., 2010; Skuladottir et al., 2016).

Supplemental vitamin D is a cost‐effective alternative to increase 25(OH)D concentrations in blood, and increases in circulating 25(OH)D with supplemental vitamin D inversely correlate with initial levels in healthy Participants (Barker et al., 2013, 2015; Trang et al., 1998). Thus, those with the lowest initial 25(OH)D generally have the greatest relative increase in circulating concentrations with supplemental vitamin D. Surprisingly, no previous study has identified if supplemental vitamin D can block the decrease in circulating 25(OH)D mediated by acute stress, and secondly, if acute stress dissociates the relationship between initial 25(OH)D concentrations and the 25(OH)D changes induced by supplemental vitamin D. It is also unknown if the decrease in circulating 25(OH)D induced by the acute stress of open‐heart surgery correlates with the initial 25(OH)D concentration before surgery.

The primary purpose of this investigation was to identify if supplemental vitamin D protects against the acute decrease in plasma 25(OH)D concentrations following open‐heart surgery and during inpatient care. We hypothesized that supplemental vitamin D prevents the immediate decline in plasma 25(OH)D concentrations induced by the acute stress of open‐heart surgery and during inpatient care. As a secondary objective, we sought to identify if open‐heart surgery alters the inverse relationship between circulating 25(OH)D changes induced by supplemental vitamin D and the baseline concentration before vitamin D supplementation. Identifying the role of supplemental vitamin D to protect against the immediate decrease in circulating 25(OH)D concentrations following open‐heart surgery could establish a cost‐effective alternative to combat the manifestation of low vitamin D levels following acute stress.

## 2 MATERIALS AND METHODS

Participants (male and female ≥18 years of age) undergoing elective open‐heart surgery at Intermountain Medical Center (Intermountain Healthcare) were screened for study participation by a designated clinical research coordinator. We excluded Participants from study participation if they were unable or refused to provide consent, had a history of daily vitamin D supplementation (≥1000 IU) during the past 3 months, had evidence of hypercalcemia (plasma >10.5 mg/dL) as determined from clinical chemistries performed as a presurgical standard of care procedure, were undergoing any scheduled cardiac surgical procedure that did not require open thoracotomy, had known allergic reactions or other intolerance to oral vitamin D, were pregnant and/or lactating women, were women of child‐bearing potential who are not using acceptable means of contraception, or were participating in an interventional study within 30 days of study enrollment. We also excluded Participants if study participation increased the risk to the patient or compromised the quality of the clinical trial as determined by the lead cardiologist (JBM). We informed Participants about the study protocol and procedures and provided both written and verbal consent before participation. The senior cardiologist, one of the co‐investigators, or a study‐designated clinical research coordinator under the supervision of the senior cardiologist performed the consent process. Study procedures were in accordance with the ethical standards of the responsible institution and approved by the Institutional Review Board at Intermountain Healthcare. This trial (ASSESS‐D) was registered on clinicaltrials.gov (NCT02460211). Participant recruitment and follow‐up was performed between March 2015 and November 2017.

### 2.1 Study design and protocol

ASSESS‐D was a single‐center, randomized (1:1), double‐blind, placebo‐controlled study conducted in patients undergoing elective open‐heart surgery. Randomization was designed by the study‐specific statistician and supervised by the designated study pharmacist. Participants were randomly assigned to one of two groups: (a) vitamin D (*n* = 75; cholecalciferol, 50,000 IU/dose) or (b) placebo (*n* = 75). Participants received supplements on three separate occasions: (a) the evening before surgery, (b) on postoperative day 1, and 3) on postoperative day 2. Each Participant received the preoperative dose orally and the postoperative doses either orally or per nasogastric tube. Placebo capsules were identical in appearance and texture to the vitamin D supplement and were prepared by the inpatient pharmacy (Intermountain Healthcare).

Following consent, we obtained blood samples from each Participant at baseline (24‐hour before open‐heart surgery), on postoperative days 1 and 2 (samples were drawn several hours after study drug was given), postoperative days 3 and 4, at discharge (generally on postoperative days 5–8), and at 6 months (±6 weeks) after surgery. We obtained the 6‐month blood sample during an elective outpatient visit. Blood samples were centrifuged and aliquoted into several different cryotubes and stored at −80°C until analysis.

The primary endpoint of this study was the change in plasma 25(OH)D concentration from baseline to postoperative day 3. As secondary endpoints, we sought to determine: (a) the correlation between serum 25(OH)D concentrations at baseline and concentration changes after surgery; and (b) if supplemental vitamin D alters inpatient and outpatient outcomes.

### 2.2 Study variables

Baseline clinical and laboratory variables were collected, including gender, age, body metrics, race, baseline vitamin D status, surgical indication, cardiovascular risk factors, baseline lipid panel, and creatinine results. Procedural and hospitalization‐related clinical variables were also collected, including the type of procedure performed, type of vascular protection offered (on‐ or off‐pump), time intubated, time in the intensive care unit, units of blood transfused, whether they received dialysis, and length of hospital stay. We identified significant in‐hospital complications, including acute renal failure (i.e., a rise in serum creatinine ≥0.5 mg/dL), stroke, myocardial infarction (as defined by new Q waves or the rise of troponin I greater than five times the upper limits of normal), the need to return to the operating room, acute heart failure, pneumonia, systemic infection, and death. Six months after surgery, each Participant returned to the clinic. Medication usage (including any vitamin D supplementation), any relevant clinical events such as the occurrence of a major adverse cardiovascular event (MACE: defined as the composite of death, myocardial infarction, stroke, repeat coronary revascularization, and hospitalization for heart failure), or a major noncardiovascular adverse event was recorded, including worsening of renal function, acute systemic infection, or hospitalization for pneumonia.

### 2.3 Plasma 25(OH)D concentrations

Total plasma 25(OH)D concentrations were measured using an enzyme‐linked immunosorbent assay (R&D Systems, RDKAP1971). All 25(OH)D testing was performed at Intermountain Healthcare's Center for Molecular and Genetic Research at LDS Hospital Cardiovascular Genetics Laboratory. Participants were classified as vitamin D deficient, insufficient, or sufficient if they had a plasma 25(OH)D concentration ≤20, 21–29, or ≥30 ng/mL, respectively (Holick et al., 2012).

### 2.4 Power calculation

The primary outcome of this study was the change in plasma 25(OH)D concentration from baseline to postoperative day 3. Data from our extensive angiographic registry (n = 4,140; Intermountain Heart Collaborative Study database) indicate that plasma 25(OH)D concentrations are ~24.2 (8.1) ng/mL in the targeted cohort. Assuming patients enrolled in this study would have similar levels, we estimated that plasma 25(OH)D concentrations would be approximately 25.0 ng/mL upon study enrollment and before supplementation. Hypothesizing an approximate 20% (Alemzadeh et al., 2012; Bang et al., 2011; Barker et al., 2012; Binkley et al., 2017; Borgermann et al., 2012; Canbaz et al., 2008; Krishnan et al., 2010; Louw et al., 1992; Reid et al., 2011; Skuladottir et al., 2016; Waldron et al., 2013) decrease in plasma 25(OH)D concentrations from baseline to postoperative day 3 in the placebo group, and setting a two‐sided α of 0.05, it was calculated that 37 Participants were required in each group to achieve a power of 90%. To account for potential dropouts, and to increase our power to perform other secondary analyses, we targeted a total of 150 patients (n = 75 per group) for study enrollment.

Statistical Analyses.

Baseline characteristics of the study participants were described using means and standard deviations for continuous variables, and proportions for discrete and categorical variables. Paired *t*‐tests and Student's t‐tests were used to compare changes in 25(OH)D levels from baseline to each follow‐up time point. Pearson's correlation coefficient was used to determine correlations between the 25(OH)D levels and the chi‐square statistic to compare differences between in‐hospital and 6‐month outcomes. All statistical analyses were performed using SPSS (version 15.0) with significance set at *p* < 0.05. All data are presented as mean (SD) unless noted otherwise.

## 3 RESULTS

### 3.1 Participant characteristics

Baseline characteristics were not significantly different between those randomized to the vitamin D or placebo group (Table 1). The plasma 25(OH)D concentration was 24.9 (16.9) ng/mL (*n* = 146), and vitamin D deficiency was found in 59 (40.4%), insufficiency in 43 (29.5%), and sufficiency in 44 (30.1%) Participants at enrollment. Thus, approximately 70% of the Participants possessed a plasma 25(OH)D concentration below the threshold for vitamin D sufficiency before supplementation. Coronary artery or valvular disease indicators for undergoing surgery were evenly distributed among groups.

**TABLE 1:** Participant characteristics at baseline

	Placebo	Vitamin D	*p*‐value
*n* (female/male)	75 (14/61)	75 (17/58)	0.55
Age (y)	62.5 (13.3)	62.4 (13.7)	0.97
Height (cm)	176 (10)	176 (12)	0.83
Body mass (kg)	92.5 (20.6)	90.8 (18.9)	0.60
Body mass index (kg/m^2^)	30.0 (7.0)	26.2 (5.4)	0.42
Creatinine (mg/dL)	1.08 (0.22)	1.06 (0.23)	0.49
eGFR (mL/min/1.73 m^2^)	72.2 (16.3)	71.9 (21.8)	0.93
Race (n)			0.79
Caucasian	72 (96.0%)	69 (92.0%)	
Other	3 (4.0%)	6 (8.0%)	
Vitamin D status (n)^a^			0.61
Sufficient	22 (30.1%)	22 (30.1%)	
Insufficient	24 (32.9%)	19 (26.0%)	
Deficient	27 (37.0%)	32 (43.8%)	
Primary surgical indication (n)			0.78
Coronary artery disease	31 (41.3%)	32 (42.7%)	
Aortic valve*	19 (25.3%)	17 (22.7%)	
Mitral valve**	7 (9.3%)	11 (14.7%)	
Aortic and mitral valves	2 (2.7%)	3 (4.0%)	
Other***	16 (21.3%)	12 (16.0%)	
Diabetes (n)	16 (21.3%)	20 (26.7%)	0.47
Smoking (n)			0.35
Current	3 (4.0%)	1 (1.3%)	
Past	16 (21.3%)	15 (20.0%)	
Never	54 (72.0%)	59 (78.7%)	
Hypertension (n)	53 (70.7%)	42 (56.0%)	0.09
SBP (mmHg)	131 (18)	129 (18)	0.45
DBP (mmHg)	77.4 (12)	76.7 (9.9)	0.68
Hyperlipidemia (n)	32 (42.7%)	38 (50.7%)	0.56
Total cholesterol (mg/dL)	170 (43)	174 (44)	0.65
HDL‐C (mg/dL)	40.4 (11.2)	42.5 (10.2)	0.32
Non‐HDL‐C^b^ (mg/dL)	129 (40)	131 (43)	0.79
LDL‐C^b^ (mg/dL)	105 (37)	109 (38)	0.63

Data are presented as mean (SD) unless otherwise noted.

Hypertension: SBP ≥140 mmHg or DBP ≥90 mmHg.

Hyperlipidemia: Total cholesterol ≥200 mg/dL, LDL ≥130 mg/dL, or on lipid lowering medication.

^a^ Plasma 25(OH)D concentration missing for four Participants (2 in each arm).

^b^ n = 110.

* Aortic insufficiency, aortic root aneurysm, aortic root replacement, bicuspid aortic valve disease.

** Mitral valve insufficiency, atrial fibrillation with chronic decompensated heart failure, rheumatic tricuspid, mitral valve disease.

*** Significant septal and apical hypertrophy, hyperlipdemia, AAA, PVI, ASD, PVR/MAZE/cardioblate, pulmonic valve regurgitation and stenosis, Marfan syndrome, aortic root dilation, PVR, septal myectomy.

### 3.2 Plasma 25(OH)D concentrations

Vitamin D status classifications (i.e., deficient, insufficient, and sufficient; Table 1) and plasma 25(OH)D concentrations (Table 2) were not significantly different between the placebo and vitamin D groups at baseline. In the placebo group, plasma 25(OH)D concentrations dropped significantly by postoperative day 1 and remained lower through postoperative day 3. However, by postoperative day 4, and thereafter, 25(OH)D concentrations began to trend toward the baseline level. By contrast, and consistent with our hypothesis, plasma 25(OH)D concentrations immediately rose by postoperative day 1, peaked at postoperative day 3, and persisted at that level throughout the duration of inpatient care in the vitamin D group. At the 6‐month follow‐up, 25(OH)D concentrations returned to levels slightly but significantly higher than that at baseline. Similar to postoperative day 3, the other secondary 25(OH)D concentration endpoints (i.e., postoperative day 1, 2, and 4, and discharge) were significantly increased, except at 6 months, in the vitamin D compared to the placebo group.

**TABLE 2:** Plasma 25(OH)D concentrations (ng/mL)

	Placebo	Vitamin D	*p*‐value
Baseline	26.5 (16.9)	23.4 (12.7)	0.37
Day 1	23.2 (11.9)	27.0 (11.7)	0.04
*p*‐value vs baseline	0.005	<0.0001	
Day 2	23.9 (12.4)	30.8 (12.0)	<0.0001
*p*‐value vs baseline	0.02	<0.0001	
*p*‐value vs day 1	0.41	<0.0001	
Day 3	23.8 (13.6)	36.0 (13.5)	<0.0001
*p*‐value vs baseline	0.01	<0.0001	
*p*‐value vs day 1	0.34	<0.0001	
*p*‐value vs day 2	0.73	<0.0001	
Day 4	25.7 (15.9)	35.4 (13.6)	0.001
*p*‐value vs baseline	0.09	<0.0001	
*p*‐value vs day 1	0.59	<0.0001	
*p*‐value vs day 2	0.75	0.0001	
*p*‐value vs day 3	0.90	0.91	
Discharge	24.5 (14.2)	36.9 (14.0)	<0.0001
*p*‐value vs baseline	0.19	<0.0001	
*p*‐value vs day 1	0.41	<0.0001	
*p*‐value vs day 2	0.82	<0.0001	
*p*‐value vs day 3	0.68	0.22	
*p*‐value vs day 4	<0.0001	<0.0001	
6 month	30.6 (17.4)	29.4 (14.3)	0.72
*p*‐value vs baseline	0.46	0.01	
*p*‐value vs day 1	0.003	0.38	
*p*‐value vs day 2	0.003	0.39	
*p*‐value vs day 3	0.009	0.001	
*p*‐value vs day 4	<0.0001	<0.0001	
*p*‐value vs discharge	0.01	<0.0001	

Data presented as mean (SD) unless otherwise noted.

### 3.3 Postoperative plasma 25(OH)D concentration changes from baseline

The change in plasma 25(OH)D from baseline to postoperative day 3 was the primary outcome of this study, and as hypothesized, it was significantly different between groups (Table 3). Similarly, changes in plasma 25(OH)D concentration were also significantly different between the placebo and vitamin D groups at postoperative days 1, 2, 4, and at discharge. Concentration changes at 6 months were not significantly different between study groups. Postoperative plasma 25(OH)D concentration changes at each time point inversely correlated with baseline 25(OH)D concentrations in both the placebo and vitamin D groups (Table 4).

**TABLE 3:** Postoperative plasma 25(OH)D concentration changes (ng/mL) from baseline

	Placebo	Vitamin D	*p*‐value
Day 1	−3.2 (9.3)	3.5 (11.0)	<0.0001
Day 2	−2.7 (9.7)	7.4 (9.0)	<0.0001
*p*‐value vs day 1	0.41	<0.0001	
Day 3	−2.8 (8.7)	12.2 (11.1)	<0.0001
*p*‐value vs day 1	0.34	<0.0001	
*p*‐value vs day 2	0.73	<0.0001	
Day 4	−3.2 (10.8)	11.7 (10.4)	<0.0001
*p*‐value vs day 1	0.59	0.003	
*p*‐value vs day 2	0.75	0.001	
*p*‐value vs day 3	0.90	0.89	
Discharge	−2.3 (14.0)	13.4 (12.3)	<0.0001
*p*‐value vs day 1	0.41	<0.0001	
*p*‐value vs day 2	0.82	<0.0001	
*p*‐value vs day 3	0.68	0.22	
*p*‐value vs day 4	0.47	0.07	
6 month	1.7 (14.7)	4.9 (12.2)	0.27
*p*‐value vs day 1	0.003	0.02	
*p*‐value vs day 2	0.003	0.39	
*p*‐value vs day 3	0.009	0.001	
*p*‐value vs day 4	0.02	0.02	
*p*‐value vs discharge	0.01	<0.0001	

Data presented as mean (SD) unless otherwise noted.

**TABLE 4:** Correlation coefficients between baseline plasma 25(OH)D concentrations (ng/mL) and postoperative concentration (ng/mL) changes from baseline in each group

Postoperative plasma 25(OH)D concentration change (ng/mL) from baseline	Baseline plasma 25(OH)D (ng/mL)	
	Placebo	Vitamin D
Day 1	−0.59^a^	−0.42^a^
Day 2	−0.59^a^	−0.38^a^
Day 3	−0.62^a^	−0.28^a^
Day 4	−0.60^a^	−0.35^a^
Discharge	−0.68^a^	−0.32^a^
6 month	−0.34^a^	−0.43^a^

^a^ *p* < 0.0001.

### 3.4 Inpatient and outpatient outcomes and serious adverse events

Inpatient outcomes and serious adverse events did not differ significantly between groups (Table 5). Outpatient outcomes at 6 months were available on 136 Participants (90.7%; placebo, *n* = 67 (89%); vitamin D, *n* = 69 (92%)) (Table 6). Six Participants voluntarily withdrew from study participation, and eight Participants were lost to follow‐up. Outpatient outcomes and serious adverse events were not significantly different between groups. However, there tended (both *p* = 0.06) to be fewer heart failure hospitalizations, and other serious adverse events (i.e., atrial fibrillation, pacemaker, cardiomyopathy) in the vitamin D group.

**TABLE 5:** Inpatient outcomes and serious adverse events

	Placebo	Vitamin D	*p*‐value
Length of stay (d)	8.0 (7.4)	8.6 (5.3)	0.35
Length of time in ICU (d)	2.9 (4.8)	3.4 (4.2)	0.22
Length of time intubated (d)	1.9 (4.2)	1.9 (3.3)	0.50
Participants transfused (n)			
Operatively	7 (9%)	16 (21%)	0.24
Postoperatively	31 (41%)	33 (44%)	0.74
Blood transfused (Units)			
Operatively	4.1 (5.8)	2.8 (1.8)	0.89
Postoperatively	4.9 (10.9)	3.0 (4.8)	0.73
Dialysis (n)	0 (0%)	1 (1.3%)	1.00
Renal failure (n)	2 (2.7%)	5 (6.7%)	0.44
Myocardial infarction (n)	4 (5.5%)	5 (6.8%)	1.00
Stroke (n)	2 (2.7%)	3 (4.0%)	1.00
Return to operating room (n)	4 (5.5%)	4 (5.3%)	1.00
New heart failure diagnosis (n)	2 (2.7%)	3 (4.0%)	1.00
Death (n)	0 (0%)	0 (0%)	—
Pneumonia (n)	1 (1.4%)	1 (1.3%)	1.00
Infection (n)	1 (1.4%)	0 (0%)	0.49

^a^ Stenosis or regurgitation.

Data presented as mean (SD) unless otherwise noted.

**TABLE 6:** Outcomes and serious adverse events at 6 months

	Placebo	Vitamin D	*p*‐value
Any serious adverse event (n)	6 (9.0%)	7 (10.1%)	0.81
Death (n)	0 (0%)	2 (2.9%)	0.50
Myocardial infarction (n)	0 (0%)	1 (1.4%)	1.00
Stroke (n)	0 (0%)	3 (4.3%)	0.25
Revascularization (n)	0 (0%)	2 (2.9%)	0.50
Heart failure hospitalizations (n)	6 (9.0%)	1 (1.4%)	0.06
Other (n)			0.06
Atrial fibrillation	2 (3.0%)	0 (0%)	
Pacemaker	1 (1.5%)	0 (0%)	
Cardiomyopathy	1 (1.5%)	0 (0%)	

## 4 DISCUSSION

This investigation provides the first evidence that supplemental vitamin D protects against the immediate reduction in circulating 25(OH)D concentrations induced by open‐heart surgery. The 25(OH)D postoperative reduction without and postoperative rise with supplemental vitamin D inversely correlated with baseline 25(OH)D concentrations, thereby indicating the importance of initial concentrations on determining the circulating vitamin D response to supplementation and as provoked by acute stress.

In addition to protecting against the reduction in postoperative 25(OH) concentrations, we also demonstrate that there was arguably an early plateau in plasma 25(OH)D concentrations from postoperative day 3 to discharge in the vitamin D group. Prior studies identify a continuous rise in serum 25(OH)D concentrations 7 to 14 days after a single bolus of cholecalciferol at 50,000 or 100,000 IU (Armas et al., 2004; Barker et al., 2017; Heaney et al., 2008; Ilahi et al., 2008). Although those findings were reported in healthy Participants (Armas et al., 2004; Barker et al., 2017; Heaney et al., 2008; Ilahi et al., 2008), we anticipated a continuous increase in plasma 25(OH)D following cholecalciferol supplementation and prior to hospital discharge in open‐heart surgery patients. This study, however, challenges this assumption and provides original data suggesting that plasma 25(OH)D concentrations reach and maintain their zenith 3 days after open‐heart surgery and only 24 hours after the last of three loading doses (50,000 IU of cholecalciferol each) of supplemental vitamin D over 4 days. An explanation for the early plateau in plasma 25(OH)D following a total of 150,000 IU of cholecalciferol supplementation, and after open‐heart surgery, awaits future resolution. It is plausible, though, that contrasting doses of supplemental vitamin D between studies, differences in the underlying pathophysiology between study cohorts, and the increased presence of potential mediators that accelerate the metabolism of circulating vitamin D in response to acute stress may contribute to the early plateau in 25(OH)D after open‐heart surgery in the vitamin D group.

As mentioned, open‐heart surgery induces acute stress (Hoda et al., 2006) and a systemic inflammatory response (Borgermann et al., 2012; Canbaz et al., 2008; Czerny et al., 2000; Holmes et al., 2002), and systemic inflammation associates with a decrease in circulating 25(OH)D (Alemzadeh et al., 2012; Bang et al., 2011; Barker et al., 2012; Binkley et al., 2017; Dahl et al., 1998; De et al., 2014; Duncan et al., 2012; Ghashut et al., 2014; Grama et al., 2020 ; Henriksen et al., 2014; Krishnan et al., 2010; Louw et al., 1992; Reid et al., 2011; Vayrynen et al., 2016; Waldron et al., 2013). Although the mechanism regulating the decrease and confounding the interpretation of endogenous vitamin D levels during inflammation is obscure, various cytokines have been found to control the enzymatic machinery that accelerates the metabolism of 25(OH)D in vitro (Edfeldt et al., 2010; Hummel et al., 2013; Koeffler et al., 1985; Pryke et al., 1990; Reichel et al., 1987; Stoffels et al., 2006, 2007) and, therefore, could account for the decrease in circulating 25(OH)D concentrations when systemic levels are elevated. Consistent with this premise, results from our laboratory imply that a pro‐inflammatory cytokine increases the most biologically active form of vitamin D (i.e., 1,25‐dihydroxyvitamin D) at the expense of compromising circulating 25(OH)D concentrations during inflammation following acute stress (Barker et al., 2012). More recently, compelling evidence by Smolders and colleagues (Smolders et al., 2020) indicate that systemic inflammation (i.e., elevation in tumor necrosis factor‐α, interleukin‐6, and interleukin‐8) following lipopolysaccharide infusion lowers circulating 25(OH)D concentrations in humans.

Similar to inflammation, other factors may contribute to the decrease in circulating 25(OH)D concentrations identified in the placebo group following open‐heart surgery. For example, oxidative stress is routinely associated with the acute stress during and following surgery, and oxidative stress or a lower antioxidant capacity decreases circulating 25(OH)D concentrations by altering the expression of genes involved with vitamin D metabolism (Abbasalizad Farhangi & Najafi, 2018; Jain et al., 2015, 2018; Parsanathan & Jain, 2019a, 2019b). Additionally, postoperative fluids administered to critically ill patients undergoing cardiopulmonary bypass surgery contributes to an acute dilution effect and lowers 25(OH)D concentrations in the blood the first 24 hours after surgery (Krishnan et al., 2010), but this may be surgery specific, as findings are not consistent with other operations where lower volumes of isotonic fluid are administered (Binkley et al., 2017; Reid et al., 2011). Hopefully, future research will reveal condition‐specific mechanisms contributing to the postoperative reduction of circulating 25(OH)D. Moreover, those endeavors will illuminate if such mechanisms that mediate a decrease in circulating 25(OH)D also hinder the efficacy of supplemental vitamin D to increase 25(OH)D in the blood during or following acute stress.

The inverse association between circulating 25(OH)D concentration changes from baseline with vitamin D supplementation and baseline 25(OH)D concentrations is well‐established (Barker et al., 2013, 2015; Trang et al., 1998). Here, we extend those findings by demonstrating that this inverse relationship persists with and without supplemental vitamin D following acute stress. Specifically, a higher initial circulating 25(OH)D concentration exhibits a smaller concentration increase to supplemental vitamin D following acute stress but a greater concentration decrease due to acute stress in the absence of vitamin D supplementation. Thus, in the absence of vitamin D supplementation, patients with a higher initial circulating 25(OH)D concentration could be predisposed to a greater concentration decrease after open‐heart surgery or other acute stressors.

Despite the rebound in plasma 25(OH)D concentrations 6 months after surgery in the placebo group, there was a trend (*p* = 0.06) for supplemental vitamin D to decrease the number of hospitalizations for heart failure or other reasons after open‐heart surgery compared to the placebo group. Still, for the most part, clinical outcomes were not significantly different between groups. It is worth noting that this study is not powered to compare outcomes or plasma 25(OH)D concentrations between groups 6 months after surgery. The data from this study, however, are essential for future hypothesis generation and sample size determinations in research investigating the influence of supplemental vitamin D on diverse clinical outcomes following open‐heart surgery. Also, in addition to the mechanisms described above, there are other factors potentially contributing to the rebound in plasma 25(OH)D concentrations in the placebo group at 6 months, including but not limited to the temporal resolution in postoperative inflammation, seasonal changes in sun exposure due to the length of the study protocol, alterations in dietary intake after surgery, and perturbations in body composition not revealed by the body mass index measure included herein.

There are limitations to this study that are worthy of discussion. First, we did not assess calcium or other mediators responsible for regulating circulating 25(OH)D due to the perioperative design of the study intervention and primary outcome. Second, evaluating other vitamin D metabolites or vitamin D carrier proteins might be advantageous to understanding the peri‐ and postoperative role of acute stress on vitamin D metabolism. Finally, to determine the potential impact of acute stress on the efficacy of supplementation to increase circulating 25(OH)D, it is necessary to compare the supplemental vitamin D‐induced 25(OH)D response with acute stress to without acute stress. Clearly, additional research identifying the mediators governing the alterations in plasma 25(OH)D with and without supplemental vitamin D following acute stress is needed.

In this trial, supplemental vitamin D was found to protect against the transient decrease in plasma 25(OH)D concentrations that occur in response to open‐heart surgery. The postoperative change in plasma 25(OH)D with and without vitamin D supplementation was found to inversely associate with initial concentrations. Therefore, we conclude that perioperative vitamin D supplementation protects against acute and transient 25(OH)D decreases mediated by open‐heart surgery. We also conclude that patients with higher baseline 25(OH)D concentrations display a smaller concentration increase to supplemental vitamin D but a greater concentration decrease in the absence of supplemental vitamin D following open‐heart surgery. The impact of vitamin D supplementation on clinical outcomes remains for larger studies to determine.

## DATA SHARING

Data described in this manuscript will not be made available because it contains potentially identifying or sensitive patient information.

## DISCLOURES

No conflicts of interest, financial or otherwise are declared by the authors.

## CONFLICT OF INTERESTS

No authors have any conflict to report.

## AUTHOR CONTRIBUTIONS

JBM designed the research; KK, BC, SI, and OG conducted the research; HTM analyzed the data; TB, HTM, and JBM wrote the paper and had primary responsibility for final content. All the authors read and approved the final manuscript.
